# An assessment of medical students’ proficiency in the diagnosis and management of snakebites: a cross-sectional study from Palestine

**DOI:** 10.1186/s12995-020-00254-3

**Published:** 2020-02-19

**Authors:** Suha S. Sulaiman, Isra K. Kharusha, Ahmad M. Samara, Samah W. Al-Jabi, Sa’ed H. Zyoud

**Affiliations:** 1 Department of Medicine, College of Medicine and Health Sciences, An-Najah National University, Nablus, 44839 Palestine; 2 Department of Clinical and Community Pharmacy, College of Medicine and Health Sciences, An-Najah National University, Nablus, 44839 Palestine; 3 Poison Control and Drug Information Center (PCDIC), College of Medicine and Health Sciences, An-Najah National University, Nablus, 44839 Palestine; 4 Clinical Research Center, An-Najah National University Hospital, Nablus, 44839 Palestine

**Keywords:** Snakebite, Medical students, Knowledge, First aid, Attitude, Diagnosis, Management

## Abstract

**Background:**

Snakebites are emergent and life-threatening injuries that may require intensive care. Physicians face difficulties in dealing with snakebite injuries due to the knowledge gaps in the diagnosis and management of snakebites. The study aimed to assess medical students’ knowledge about the diagnosis and management of snakebite injuries, as well as their proficiency in first aid methods in case of snakebite and perception regarding snakes and snakebite injuries.

**Methods:**

A cross-sectional study conducted among 200 medical students in their clinical years at An-Najah National University. A questionnaire was developed and distributed among those students. The questionnaire assessed the students’ knowledge regarding the diagnosis and management of snakebites and their attitude regarding snakes and snakebites. The total scores of knowledge were obtained and tested based on the participants’ demographic characteristics using the Kruskal-Wallis test and the Mann-Whitney U test. *P*-values of < 0.05 were considered to be statistically significant.

**Results:**

The mean age of participating medical students was 22.2 ± 2.4 (year). Half of these medical students were in there final year of study (sixth year). After the analysis was done, we found a knowledge deficit in snakebite diagnosis and management among medical students. The mean knowledge scores regarding *Vipera palaestinae*, signs and symptoms, laboratory investigations, anti-venom, and first aid were 3.8/13, 8.2/16, 6.1/10, 3.6/11 and 8.3/15 respectively for medical students. It was found that medical students in higher years of study had a higher knowledge of laboratory investigation, and males were more knowledgeable in the correct way for first aid methods than females (*p* < 0.036).

**Conclusions:**

The level of knowledge regarding the diagnosis and management of snakebites was not good enough among most of the students. In order to improve their knowledge, snakebite diagnosis and management should be introduced and focused on in medical curriculum. Also, formal first aid training classes for medical students should be introduced in order to teach them the correct and updated methods of first aid as they will be the future health care providers and proper first aid will effectively decrease morbidity and mortality of snakebites.

## Background

Snakebites are emergent and life-threatening injuries that may require intensive care. Physicians face difficulties in dealing with snakebite injuries due to the knowledge gaps in the diagnosis and management of snakebites, including the administration of anti-venom therapy, which in turn is due to the lack of clinical practice [1, 2].

There are no diagnostic markers or kits available to definitely diagnose envenomation in clinical practice. Therefore, identification of the snake and observation of the clinical signs and symptoms of envenomation are required in order to be able to diagnose snakebite envenoming [1].

During the treatment of snakebites, the priority is to transport the victim as quickly as possible to medical care and try to delay life-threatening shock and respiratory paralysis until professional care is available [3, 4]. In most tropical developing countries [2, 3, 5], topical herbs, traditional remedies, incisions, snake heads, ligatures and other dangerous techniques are often the immediate treatment of snakebites. These unsuitable techniques delay presentation, distort the clinical picture, and can lead to complications such as bleeding, infection, and gangrene. In order to educate affected communities, modern methods of health promotion should be used [3]. This can be achieved by encouraging swift transport to medical care and discouraging the ineffective and harmful traditional treatments. Some first aid techniques are difficult to use in practice in developing countries [6]. Therefore, the use of other measures such as the early administration of anti-venom, quick transportation of victims to medical care, and educating the medical teams on proper first aid measures for the resuscitation of victims are preferred.

The Global Burden of Disease 2016 study estimated that the total death from snakebite envenomation approached 79,000. The estimated number of people who face permanent disabilities, including restricted mobility, extensive scarring and contractures, blindness, and amputation following snakebite envenomation is 400,000 a year [7]. These public health data indicate that the global burden of snakebite envenomation, measured by incidence, mortality, and economic costs is high. Therefore, learning the proper methods of diagnosis and management is of great importance.

Many countries, especially ones that have high incidence rates of snakebites, have implemented effective first aid management methods to help decrease morbidity and mortality rates among patients affected by snakebite. The most emphasized method was ensuring quick transport of the patients to the closest healthcare center that have both well-trained personnel and anti-venom [8]. Despite these efforts, many improper first aid methods are still being used in many countries [9–11].

In Palestine, it was estimated that 100–150 people per year experience venomous bites, and about 2–3 of them die. Venomous snakes found in Palestine include Palestine saw-scaled viper (*Echis coloratus*), Palestine viper (*Vipera palaestinae*), Desert Black Cobra (Walterinnesia aegytia) and Palestine Mole Viper (Atractaspis engaddensis) snakes among others [12]. The Palestinian viper (*Vipera palaestinae*) is the most common and dangerous venomous snake in the Middle East. It is also responsible for most envenomation in humans in Palestine [13]. It has the ability to bite the victim rapidly without warning. The fatality rate for envenomation of *Vipera palaestinae* is about 5% even with anti-venom administration [14]. According to the Palestinian economy portal, the Palestinian Ministry of Health (MOH) pays around 5 million ILS (around 1.4 million USD) every year to procure the anti-venom for the *Vipera palaestinae* snake. Each victim costs the MOH around 24,500 NIS (around 6600 USD) [15].

The study aimed to assess medical students’ knowledge about the diagnosis and management of snakebite injuries. It also assesses their proficiency in first aid methods in case of snakebite and perception regarding snakes and snakebite injuries. The present research explores, for the first time in Palestine, this area of medical knowledge, and its results provide an important guide to improve the quality of medical education by identifying gaps in the curricula of medical school and related fields. Also, identifying such gaps and taking the proper actions to address them will subsequently have a positive effect on patients care and public health in general.

## Methods

### Study design

This was a cross-sectional study targeting medical students in An-Najah National University (ANNU).

### Study setting

This study was conducted in ANNU, which is located in Nablus city (the second largest city in the West Bank) in the north of Palestine.

### Study population and sample size

Two hundred medical students from ANNU were selected for the sample of this study.

### Inclusion and exclusion criteria

The Inclusion criteria for this study included being a student enrolled in ANNU, being a medical student, and being at least in the fourth year. Students in basic years (first, second, and third years) or from specialties other than medicine were excluded.

### Data collection form

We used a questionnaire that tested knowledge on proper first aid methods to deal with snakebites among the participants. It was designed around the World Health Organization (WHO) protocol for managing snakebites and benefited from previous related studies [8, 9, 12, 13, 16–28].

The data collection instruments contained eight sections:

- The first section presented questions on specific demographic data, including age, gender, place of residence, and educational level.
- The second section included 10 items on self-evaluation regarding snakebites. These items inquired about self-assessment of the participant’s knowledge (good, average, or poor), the need for knowledge regarding snakebite (high, moderate, or low), whether the participant or a member of the participants’ family experienced a snakebite before, whether she or he received training on snakebite management and whether they believed there is a need for such training, the adequacy of the health facility to deal with snakebites as perceived by the participant, the source of his or her knowledge in snakebite management (medical education, television, written media, relatives, and/or the internet), and her of his first reaction in the case of snakebite.
- The third section assessed the knowledge scale regarding *Vipera palaestinae* snake, which is the most commonly encountered venomous snake in Palestine. This section consisted of 13 true or false questions, and the participants received one point for each correct answer. A total score for this section was generated by calculating the total number of points achieved by the participant.
- The fourth section assessed the knowledge scale regarding the general signs and symptoms of venomous snakebites. In this section, we listed 16 possible signs/symptoms of venomous snake bites and asked the participants to mark all the possible signs and symptoms of snakebites from the list. The number of signs/symptoms the participants identified from the list represented their total score on this section.
- The fifth section assessed the knowledge scale regarding laboratory tests following snakebites. This section consisted of a list of 10 laboratory tests that should be performed on a snakebite victim, and we asked the participants to identify all the necessary laboratory tests in cases of snakebite. The number of the selected tests represented the participant’s total score in this section.
- The sixth section assessed the knowledge scale regarding anti-venom preparation and administration after a snakebite and consisted of three parts. The first part contained 2 true or false items. The second part contained 6 multiple-choice items, each with 3 possible options, as well as an additional (I do not know) option. The third part listed 3 possible complications of anti-venom administration and asked the participant to mark all the possible complications of anti-venom administration from the list. The participant received one point for each correct answer in the first and second parts and one point for each complication they identified from the third-part. The total sum of the points they received represented their total score on this section.
- The seventh section assessed the knowledge scale regarding first aid management in cases of snakebite through 15 items. Each item presented a statement and the participant was asked to answer with true, false, or I do not know for each statement. The participant received one point for each correct answer and the total sum of points represented his or her total score on this section.
- The eighth and final section addressed the participants’ general attitudes towards snakes and snakebites. In this section, the participant was presented with 8 statement on the proper attitude towards snakebites, and was asked to mark each statement as true or false or answer with I do not know. The participant then received one point for each correct answer and the total sum of points she or he achieved was calculated, which also represented the total score for this section.

### Validity and reliability study

A panel of three qualified clinical pharmacologists and pharmacists who are specialists in this type of studies reviewed and evaluated the final questionnaire’s face and content validity, and assessed the organisation, definition of words, medical terminology, appropriateness, completeness, and a clear sequence of statements. After finalizing the questionnaire, the reliability and clarity of the questions was pre-tested among 10 medical students, and the necessary changes were made accordingly. The final analysis did not include these 10 medical students. In order to assess the questionnaire’s reliability, internal consistency testing was performed. Cronbach’s alpha was used to determine the internal consistency. Cronbach’s alpha coefficients were acceptable for knowledge scale regarding *Vipera palaestinae* (0.819), knowledge scale regarding the general signs and symptoms of venomous snakebite (0.900), knowledge scale regarding laboratory test following snakebite (0.866), knowledge scale regarding antivenom (0.827), knowledge scale regarding first aid management (0.847), and attitudes scale toward snakes and snakebite (0.718).

### Ethical issue

Institutional Review Board (IRB) approval from ANNU was granted before conducting this study. The questionnaire was distributed to students after their lectures or between the lectures after they verbally consented to take part in this research. The purpose of the study was clarified and students were encouraged to answer questions accurately and objectively as best as they can. It was also made clear to the participants that the collected data was used for proposes of research only and without any identifiers.

### Statistical analysis

Data analysis was carried out using IBM Statistical Package for the Social Sciences (SPSS) Version 21. Data expressed as means ± SD for continuous variables and as frequencies (percentage) for categorical variables. Variables that were not normally distributed were expressed as medians (lower–upper quartiles). Variables were tested for normality. Chi-square, Mann-Whitney U test, and Kruskal-Wallis test were used to test the difference between different categories as appropriate. We considered the *p*-value as statistically significant at < 0.05 level.

## Results

### Demographic data

There were 200 questionnaires distributed among medical students who are in their clinical years at ANNU. The mean age of the participating students was 22.2 (year) and the standard deviation (SD) was 2. Of the total 200 students participating in the study, male students accounted for 45%. The majority (60.5%) of students lived in a city, and there were 101 students (50.5%) at their sixth year of study, 34 (17%) in their fifth year, and 65 (32.5%) in their fourth year as shown in Table 1.

**Table 1:** Demographic characteristics of medical student

Characteristic	Number (%), *N* = 200
Gender	
Male	90 (45)
Female	110 (55)
Residency	
City	121 (60.5)
Village	77 (38.5)
Palestinian refugee camp	2 (1)
Year of study	
Fourth year	65 (32.5)
Fifth year	34 (17)
Sixth year	101 (50.5)

### Self-evaluation

As shown in Table 2, 112 of the participants (56%) rated their knowledge as poor, 65 (32.5%) rated their knowledge as average and 23 (11.5%) rated their knowledge as good. Regarding the demand for knowledge on snakebites, 105 of the participants (52.5%) rated their demand for knowledge as high, 74 (37%) rated it as moderate, and 21 (10.5%) rated it as low. Most of medical students 187 (93.5%) think that there is a need for training on snakebite management, and only 27 (13.5%) reported that they were trained to deal with snakebite patient. A majority of 143 participants (71.5%) thought that there are adequate facilities in the hospitals for snakebite management. Most of the participants obtained their knowledge on snakebite management from television (30%) or the internet (29.5%). Only 41% of participants reported that they would call a medical professional if they faced snakebite whereas 40% reported that they would take simple intervention immediately, and 19% reported that they would be too nervous to do anything.

**Table 2:** Medical students’ responses to self-assessment questions

Question	Number (%)
“How would you rate your knowledge about snakebite?”	
Good	23 (11.5)
Average	65 (32.5)
Poor	112 (56)
“How would you rate your demands for knowledge about snakebite?”	
High	105 (52.5)
Moderate	74 (37)
Low	21 (10.5)
Have you ever experienced snakebite?	
Yes	4 (2)
No	196 (98)
Have a member of your family ever experienced snakebite?	
Yes	7 (3.5)
No	193 (96.5)
Did you receive training about dealing with snakebite patient?	
Yes	27 (13.5)
No	173 (86.5)
Do you think that there is a need for training on snakebite management?	
Yes	187 (93.5)
No	13 (6.5)
Do you think that there is an adequate facility in hospitals for snakebite management?	
Yes	143 (71.5)
No	57 (28.5)
Where did you obtain the knowledge of snakebite?	
Medical education	45 (22.5)
Television	60 (30)
Books/magazine/newspapers	24 (12)
Families/friends	12 (6)
Internet	59 (29.5)
“What is your first reaction will be if you face snakebite?”	
Too nervous to do anything	38 (19)
Call for surgeon or medical colleague	82 (41)
Take simple intervention immediately	80 (40)

### Knowledge on the diagnosis and management of snakebite

The average score of the participants was 3.8 points (the highest possible score was 13 points) for items on knowledge regarding *Vipera palaestinae*, with a SD of 2.6. It was observed that most of participants (62.5%) knew that *Vipera palaestinae* is the most common venomous snake in Palestine. Only 10% believed that the mortality rate due to *Vipera palaestinae* envenomation is 1.5%. Only 1.5% of the students knew that total serum cholesterol level on admission may serve as an indicator of the severity of envenomation. The percentages of correct responses of medical students to questions on knowledge regarding Palestinian vipera snake are shown in Table 3.

**Table 3:** Percentages of correct responses of medical students to questions on knowledge regarding *Vipera palaestinae* snake

Question	Number (%)
“*Vipera palaestinae* is the most common venomous snake in Palestine” (T)	125 (62.5)
“Its length is 1 m in average” (T)	73 (36.5)
“The mortality rate due to Vipera palaestinae envenomation is 1.5%” (T)	20 (10)
“Vipera palaestinae toxin contain neurotoxin compound” (T)	107 (53.5)
“Vipera palaestinae toxin contain hemorrhagic compound” (T)	45 (22.5)
“Vipera palaestinae toxin contain integrin inhibitors compound” (T)	76 (38)
“Vipera palaestinae toxin contain cardiotoxin compound” (T)	15 (7.5)
“Vipera palaestinae toxin contain growth factors inhibitors compound” (T)	12 (6)
“The most frequent clinical sign of envenomation is local swelling” (T)	93 (46.5)
“The most frequent clinical sign of envenomation is increased body temperature” (F)	26 (13)
“Envenomation were reported only in human” (F)	22 (11)
“Admission total serum cholesterol level may serve as indicator of severity of envenomation” (T)	3 (1.5)
“Males are bitten twice as often as females” (T)	74 (37)

Table 4 shows the percentages of correct answers regarding the general signs and symptoms of venomous snakebite. The mean score of the participants’ on this subject was 8.2 out of maximum 16 points with SD of 4.2. Most of the students recognized swelling with pain and blistering (75%), convulsions (79%), dizziness and vomiting (75.5%), severe muscle pain (70%), blurring of vision (67%), and shock (67%) as signs and symptoms of envenomation. Only a minority of the students recognized weakness of the neck muscle and scanty or no urine output (27%) as signs and symptoms of envenomation.

**Table 4:** Percentages of medical students who recognized signs and symptoms of snakebite^a^

Sign/ symptom	Number (%)
Swelling with pain and blistering	150 (75)
Dizziness and vomiting	151 (75.5)
Blurring of vision	134 (67)
Convulsion	158 (79)
Unconsciousness	118 (59)
Heaviness of eyelids	78 (39)
Weakness of neck muscle	54 (27)
Difficulty in swallowing	73 (36.5)
Nasal regurgitation/voice	62 (31)
Difficulty in respiration	140 (70)
Bleeding from gum and vomiting	68 (34)
Persistent bleeding from bite site	57 (28.5)
Severe muscle pain	140 (70)
Dark colored urine	64 (32)
Scanty or no urine output	54 (27)
Shock/collapse	134 (67)

^a^Questions were adapted from previous study [19]

Table 5 shows the percentages of correct responses of participants regarding laboratory test following snakebite. The average score of the participants’ knowledge about laboratory investigations was 6.1 out of maximum 10 points with SD of 2.9. Complete blood count (mentioned by 88%), 20-min whole blood clotting (73.5%), and electrolytes (71.5%) were the most frequent lab investigations mentioned by the participants.

**Table 5:** Percentages of medical students who acknowledged the necessity of certain laboratory tests following snakebite

Test	Number (%)
20-min whole blood clotting	147 (73.5)
Complete blood count	176 (88)
Blood urea	100 (50)
Creatinine	112 (56)
Electrolyte	143 (71.5)
Serum CPK	119 (59.5)
urine R/E	94 (47)
ECG	141 (70.5)
Immunodiagnosis	68 (34)
Blood grouping and Rh typing	125 (62.5)

The average score for items on anti-venom knowledge was 3.6 points (the highest possible score was 11 points) with an SD of 2.2. Table 6, section A shows the rates correct responses of medical students regarding their knowledge about the preparation of anti-venom injection. A third of them thought that there is a need to dilute anti-venom before giving it. Almost half of them (53.5%) agreed that the required amount of anti-venom vary with the severity of envenomation. Students’ rates of correct answers on the correct amounts of anti-venom for minor and major bites and student’s responses are shown in section B, and section C shows the rate of correct responses regarding participant’s knowledge of complications of anti-venom.

**Table 6:** Percentages of correct responses of medical students to questions on knowledge regarding anti-venom

Item	Number (%)
**A**	
Do you think that there is a need to dilute anti-venom before giving it?	67 (33.5)
Do you think that required amount of anti-venom vary with the severity of envenomation?	107 (53.5)
**B**	
What is the best route to give anti-venom injection?	
Intravenous (IV) (Correct answer)	109 (54.5)
Intramuscular (IM)	32 (16)
Did not know	59 (29.5)
If the patient has been envenomated, how many vials at least must be present?	
10	26 (13)
15 (Correct answer)	17 (8.5)
20	16 (8)
Did not know	141 (70.5)
How many vials should be administrated initially?	
1	24 (12)
2 (Correct answer)	22 (11)
4	14 (7)
Did not know	140 (70)
What do you think about the rate of infusion of each vial?	
One vial per minute	15 (7.5)
One vial per 15 min (Correct answer)	22 (11)
One vial per 30 min	10 (5)
Did not know	153 (76.5)
The required amount of anti-venom for a minor bite with envenomation?	
1–2	18 (9)
2–4 (Correct answer)	13 (6.5)
5–15	7 (3.5)
Did not know	162 (81)
The required amount of anti-venom for moderate or severe bites with envenomation	
1–2	3 (1.5)
2–4	11 (5.5)
5–15 (Correct answer)	24 (12)
Did not know	162 (81)
**C**	
Early anaphylaxis (urticarial, dyspnea, hypotension)	141 (70.5)
Diarrhea and vomiting	100 (50)
Pyrogenic reaction (fever and chill)	105 (52.5)

The responses of students regarding knowledge on first aid in case of snakebite are shown in Table 7. The average participant’s first aid score was 8.3 out of maximum 15 points with a SD of 3.4.

**Table 7:** Percentages of correct responses of medical students to questions on knowledge regarding first aid in case of snakebite

Question^a^	Number (%)
“Is telling the victim to stay calm beneficial?” (Yes)	180 (90)
“Should the site of the bite be raised above the level of the person’s heart?” (No)	93 (46.5)
“Should local incisions or pricks/punctures be made over the bite site?” (No)	94 (47)
“Should the wound of bite site be rinsed (not scrubbed) with water as soon as possible?” (Yes)	128 (64)
“Should healthy volunteer suck the venom out of the wound?” (No)	135 (67.5)
“Should tight bands (tourniquets) be applied around the limb proximal to the bite site?” (No)	54 (27)
“Should pressure immobilization bandages be applied around the bite site?” (Yes)	98 (49)
“Is electric current at the site of bite useful?” (No)	87 (43.5)
“Is topical instillation or application of herbs beneficial?” (No)	89 (44.5)
“Is application of ice pack at the site of bite beneficial?” (No)	30 (15)
“Is application of alcohol at the site of bite beneficial?” (No)	47 (23.5)
“Should massage of bite wound be done?” (No)	121 (60.5)
“Should the snakebite patient be transported to the hospital soon after the bite?” (Yes)	177 (88.5)
“Can envenomation be cured by anti-venom therapy?” (Yes)	160 (80)
“Are all snakebites associated with envenomation?” (No)	163 (81.5)

^a^These questions were adapted from previous studies [18, 22]

### Attitudes of students regarding snakes and snakebite

Table 8 shows the responses of the participating medical student on their attitude towards snakebite. The participants’ average score for items on the proper attitude towards snakes and snakebite was 4.5 (the highest possible score was 8 points) with an SD of 2. About 16.5% of the students incorrectly thought that the offending snake must be killed as soon as possible, whereas 15% were convinced that a snake can remember people who attack it and will seek revenge in the future. Most of the students (68%) agreed that when handling dead snakes, people may suffer venom injection by an accidental scratch from the fang of a snake’s severed head. Only 11.5% of the students thought that snakes have attractive appearance and movement patterns and are considered an important component of biodiversity. Most of the students (76%) agreed that snake venoms have medicinal value and that snakes are important for education.

**Table 8:** Percentages of correct responses of medical students to questions on attitude towards snakebite

Variable^a^	Number (%)
“The snake should be killed as far as possible after it bites the victim” (F)	33 (16.5)
“The snake will capture the image of the offender who teases it and takes revenge later” (F)	29 (15)
“When handling dead snakes, people may suffer venom injection by an accidental scratch from the fang of a snake’s severed head” (T)	136 (68)
“The venomous snake head is usually oval shaped, with regular teeth marks” (F)	68 (34)
“Snakes have attractive appearance and movement patterns; snakes are important component of biodiversity” (T)	23 (11.5)
“Snake balances natural ecosystem and contribute to food-web, prevent environmental pollution and absorbing poison from environment” (T)	96 (48)
“Some snakes are venomous and other is non- venomous, snakes do not bite until teasing” (T)	35 (17.5)
“Snake venoms have medicinal value, snakes are important for education” (T)	152 (76)

^a^These questions were adapted from previous studies [18, 20, 22]

### Comparisons of demographic characteristics of participants and knowledge scores

Table 9 shows the relationship between the knowledge score regarding Palestinian vipera snake and characteristics of the participants. Whereas the association between knowledge score on the signs and symptoms and the demographic data on the participating students are shown in Table 10.

**Table 9:** Demographic characteristics of medical students and knowledge score of Palestinian vipera snake

Characteristic	Median ^a^ [Q1-Q3]	*P* value
Gender		
Male	4 [2–6]	0.205^b^
Female	3 [1–6]	
Residency		
City	3 [1–6]	0.089^c^
Village	4 [2.5–6]	
Palestinian refugee camp	5 [3-]	
Year of study		
Fourth year	4 [2–6]	0.391^c^
Fifth year	2.5 [1–6]	
Sixth year	4 [2–6]	

^a^knowledge score regarding Palestinian vipera snake was a range from 0 to 13; high score reflects more knowledge about Palestinian vipera snake

^b^statistical significance of differences calculated using the Mann-Whiney U test

^c^statistical significance of differences calculated using the Kruskal-Wallis test

**Table 10:** Demographic characteristics of medical students and signs and symptoms score

Characteristic	Median^a^ [Q1-Q3]	*P* value
Gender		
Male	8 [6–12]	0.181^b^
Female	8 [5–10]	
Residency		
City	7 [5–11]	0.217^c^
Village	9 [6–12]	
Palestinian refugee camp	11.5 [11-]	
Year of study		
Fourth year	8 [6–10.5]	0.219^c^
Fifth year	9 [6.75–12]	
Sixth year	8 [5–11]	

^a^knowledge score of signs and symptoms of snakebites was a range from 0 to 16; high score reflects more knowledge about signs and symptoms

^b^statistical significance of differences calculated using the Mann-Whiney U test

^c^statistical significance of differences calculated using the Kruskal-Wallis test

Table 11 shows the association between knowledge score regarding laboratory investigations for snakebite s and characteristics of participants, wherein being in a higher year of study was the only factor associated with scoring higher (*p* < 0.05).

**Table 11:** Demographic characteristics of medical students and laboratory investigations score

Characteristic	Median ^a^ [Q1-Q3]	*P* value ^b^
Gender		
Male	7 [4–9]	0.416^c^
Female	6 [4–8]	
Residency		
City	6 [4–8]	0.079^d^
Village	7 [5–9]	
Palestinian refugee camp	3.5 [2-]	
Year of study		
Fourth year	5 [3–7]	**0.014**^d^
Fifth year	7 [5–9]	
Sixth year	7 [5–9]	

^a^knowledge score of laboratory investigation for snakebites was a range from 0 to 10; high score reflects more knowledge about laboratory investigations

^b^The *p*-value is bold where it is less than the significance level cut-off of 0.05

^c^Statistical significance of differences calculated using the Mann-Whiney U test

^d^Statistical significance of differences calculated using the Kruskal-Wallis test

Table 12 shows the relationship between knowledge score on anti-venom and the participants’ characteristics.

**Table 12:** Demographic characteristics of medical students and anti-venom score

Characteristic	Median ^a^ [Q1-Q3]	*P* value
Gender		
Male	3 [2–5]	0.194^b^
Female	4 [2–5]	
Residency		
City	4 [2–5]	0.354^c^
Village	4 [2–5]	
Palestinian refugee camp	5.5 [5-]	
Year of study		
Fourth year	4 [2–5]	0.412^c^
Fifth year	3 [2–5]	
Sixth year	4 [2–5]	

^a^knowledge score of anti-venom was a range from 0 to 11; high score reflects more knowledge about anti-venom

^b^statistical significance of differences calculated using the Mann-Whiney U test

^c^statistical significance of differences calculated using the Kruskal-Wallis test

Finally, the relationship between knowledge score on first aid and demographic characteristics of participants is shown in Table 13, wherein male gender was significantly associated with a higher knowledge score on first aid (*p* = 0.036).

**Table 13:** Demographic characteristics of medical students and first aid score

Characteristic	Median ^a^ [Q1-Q3]	*P* value ^b^
Gender		
Male	9.5 [7–11]	**0.036**^**c**^
Female	8 [6–11]	
Residency		
City	9 [6–11]	0.946^d^
Village	9 [6–11]	
Palestinian refugee camp	8 [7-]	
Year of study		
Fourth year	9 [6–11]	0.214^d^
Fifth year	7.5 [6–9]	
Sixth year	9 [6–12]	

^a^knowledge score of first aid was a range from 0 to 15; high score reflects more knowledge about first aid

^b^The *p*-value is bold where it is less than the significance level cut-off of 0.05

^c^Statistical significance of differences calculated using the Mann-Whiney U test

^d^Statistical significance of differences calculated using the Kruskal-Wallis test

### Comparison of demographic characteristic of participants and attitude score

Table 14 presents the association between attitude score and the participants’ demographics. Male gender showed a positive association with the attitude score (*p* < 0.001).

**Table 14:** Demographic characteristics of medical students and attitude score

Characteristic	Median ^a^ [Q1-Q3]	*P* value ^b^
Gender		
Male	5 [4–7]	**< 0.001**^**c**^
Female	4 [3–5.25]	
Residency		
City	4 [3–6]	0.307^d^
Village	5 [3.5–6]	
Palestinian refugee camp	3.5 [2-]	
Year of study		
Fourth year	5 [2.5–6]	0.878^d^
Fifth year	5 [3.75–6]	
Sixth year	5 [3–6]	

^a^Attitude score was a range from 0 to 8; high score reflects higher attitude regarding snake and snakebites

^b^The *p*-value is bold where it is less than the significance level cut-off of 0.05

^c^Statistical significance of differences calculated using the Mann-Whiney U test

^d^Statistical significance of differences calculated using the Kruskal-Wallis test

### Correlations between scores

We found weak but significant positive correlation between knowledge score of Palestinian vipera snake and knowledge score on signs and symptoms (*p* < 0.001, *r* = 0.388). Knowledge score of Palestinian vipera snake was also positively correlated with laboratory investigations score (*p* < 0.001, *r* = 0.323), anti-venom score (*p* < 0.001, *r* = 0.418), first aid score (*p* < 0.001, *r* = 0.433), and attitude score (*p* < 0.001, *r* = 0.266). Another weak but significant positive correlation was found between signs and symptoms score on one hand and laboratory investigations score (*p* < 0.001, *r* = 0.543), anti-venom score (*p* < 0.001, *r* = 0.412), first aid score (*p* < 0.001, *r* = 0.296), and attitude score (*p* < 0.001, *r* = 0.259) on the other.

Also, we found a week but significant positive correlation between laboratory investigations score and anti-venom score (*p* < 0.001, *r* = 0.449), first aid score (*p* < 0.001, *r* = 0.347), and attitude score (*p* = 004, *r* = 0.203). There is a weak but significant positive correlation between anti-venom score and each of first aid score (*p* < 0.001, *r* = 0.480) and attitude score (*p* = 0.002, *r* = 0.223). Finally, there is a weak but significant positive correlation between first aid score and attitude score (*p* < 0.001, *r* = 0.452).

## Discussion

In this cross-sectional questionnaire-based study, we interviewed 200 medical students in their clinical years (fourth, fifth, and sixth years) at ANNU. In this study we aimed to evaluate the level of knowledge among medical students on the diagnosis and management of snakebites through assessing their knowledge regarding Palestinian Vipera snake (which is the most common venomous snake in Palestine), signs and symptoms of snakebites, the necessary laboratory investigations, and anti-venom administration. This study also evaluated the students’ knowledge on first aid practices when dealing with a case of snakebite and the students’ perception regarding snakes and snakebites.

The majority of medical students (56%) rated their knowledge about snakebite as poor, and 52.5% of them rated their need to learn about snakebites as high. This indicates that knowledge about snakebites was inadequate among the participants. Only 22.5% of students obtain the knowledge of snakebites through medical education which reflects the need for more focus on teaching the students the correct and updated methods of diagnosing and managing snakebites. Most students (93.5%) thought that they need training on snakebites management and only 13.5% of the students received such training, which also indicates the need for training programs on how to deal with snakebites.

We found a serious knowledge deficit regarding the diagnosis and management of snakebites evident by the average score of *Vipera palaestinae* knowledge (3.8 out of maximum of 13).

This knowledge deficit was less prominent among medical students in their fifth and sixth year compared to those in their fourth year (*p* < 0.05). This contrast may be due to the higher likelihood of witnessing or dealing with snakebites as they advance in the clinical years as well as higher likelihood of reading and learning more about snakebite injuries.

The results of signs and symptoms and laboratory investigations knowledge in this study compares to those of a study done in Bangladesh [20].

We also found a serious deficit in anti-venom knowledge, evident by the average score was 3.6 out of maximum 11, as well as in first aid knowledge in case of snakebite (the mean score was 8.3 out of maximum 15). These knowledge gaps are more prominent among female medical students compared to male students (*p* < 0.05) which could be related to the female’s negative perception regarding snakes and snakebites which is also more prominent compared to male students (*p* < 0.05). This theory could be further supported by the presence of a moderate but significant positive correlation between first aid score and attitude score (*p* < 0.05, *r* = 0.534) among the participants. The results on first aid knowledge in this study also compared to results of other studies done in Nepal [16] and Cameron [21].

In order to improve knowledge of medical students regarding the diagnosis and management of snakebites, many courses, including internal medicine, general surgery and emergency medicine, can be supplemented by materials that focus on how to investigate, diagnose, and manage snakebites.

Organizing training courses could also help in improving knowledge regarding snakebite diagnosis and management and encourage evidence-based first aid practices, as evident from a study done among health worker in Cameron [21].

However, the higher level of knowledge necessary for professional management would be hard to retain throughout the physician’s professional career, considering the rarity of snakebite envenoming as encountered by Palestinian physicians (2–3 cases per 100,000 per year). So, in addition to learning basic knowledge about snakebite envenoming, it is important to have a toxicology center that instantly provides doctors with information about the management of snakebite envenoming from a specialist around the clock. In Palestine, the only such facility, the Poison Control and Drug Information Center (PCDIC), was established at ANNU in 2006 with the primary goal of providing information to the general public or medical institutions and healthcare professionals who are in urgent need of information about poisoning and envenoming [29, 30].

However, for the PCDIC to be effective in achieving its goal, better coordination and communication between PCDIC and Palestinian hospitals should be established. This should include periodic reporting on the type and quantity of antidotes in each hospital to direct snakebite envenoming patients to hospitals where appropriate management resources are available. It is also important to encourage doctors and healthcare professionals to contact the PCDIC whenever its services are needed.

### Strength and limitations

This was the first study to assess the knowledge about snakebites and first aid measures among medical students in Palestine. The study showed a lack of knowledge among students on these subjects, which emphasizes the need for solutions. Also, the presence of multiple cases of snakebite envenomation every year in Palestine highlights the importance of adequate knowledge on how to deal with these cases, which is one of the objectives of this study. Also, this research could encourage more studies to look into the knowledge and practical skills among medical students and doctors.

Among its limitations, this study assessed students’ knowledge about how to deal with snakebite cases theoretically but did not assess their first aid skills in a practical way. Another limitation is that this study was conducted in one university; therefore its findings cannot be generalized to all universities in different parts of Palestine**.**

## Conclusions

In conclusion, the study showed that there is a lack of knowledge about the methods of dealing correctly with snakebite cases and the lack of appreciation of the importance of this subject. We recommend a greater focus on this subject because students are likely to encounter such cases in their professional and practical life. The study also identified key areas in which knowledge was lacking. More studies should also evaluate the knowledge and skills of first aid among health-care providers in Palestine.

## Data Availability

The datasets used and/or analyzed during the current study are available from the corresponding author on reasonable request.

## Abbreviations

ANNU: An-Najah National University
ILS: Israeli new shekel
IRB: Institutional Review Board
MOH: Ministry of Health
PCDIC: Poison Control and Drug Information Center
SD: Standard deviation
USD: United States Dollar
WHO: World Health Organization
